# Leveraging mHealth and Patient Supporters for African Americans' and Latinxs' Engagement in HIV Care (LEAN): Protocol for a Randomized, Controlled, Effectiveness-Implementation Trial

**DOI:** 10.2196/42691

**Published:** 2023-02-14

**Authors:** Joyce Jones, Jane McKenzie-White, Ronald Saxton, Suzanne M Grieb, Bareng Nonyane, Cadeesha Graham, Anthony Cano, Sheridan Johnson, Lanisha Childs, Adena Greenbaum, Colin Flynn, Marcia Pearlowitz, Shivaun Celano, Larry W Chang, Kathleen R Page

**Affiliations:** 1 School of Medicine Johns Hopkins Baltimore, MD United States; 2 Bloomberg School of Public Health Johns Hopkins Baltimore, MD United States; 3 Baltimore City Health Department Baltimore, MD United States; 4 Maryland Department of Health Baltimore, MD United States

**Keywords:** mHealth, HIV linkage to care, HIV virologic suppression, HIV continuum of care, adherence, implementation

## Abstract

**Background:**

Despite substantial investments in ending the HIV epidemic, disparities in HIV care persist, and there is an urgent need to evaluate novel and scalable approaches to improving HIV care engagement and viral suppression in real-world settings.

**Objective:**

This paper aims to describe a study protocol for a pragmatic type II hybrid effectiveness-implementation randomized controlled trial comparing existing standard of care clinic HIV linkage, adherence, and retention (LAR) protocols to a mobile health (mHealth)–enhanced linkage, adherence, and retention (mLAR) intervention.

**Methods:**

The study will enroll 450 participants from clinics in Baltimore City. Eligibility criteria include being ≥18 years of age, having a new HIV diagnosis or being HIV-positive and out of care, or being HIV-positive and deemed by clinic staff as someone who could benefit from linkage and retention services. Participants randomized to the intervention receive mHealth-supported patient navigation for 12 months. Participants in the control group receive the referring clinic’s standard of care patient support. The primary outcome is HIV virologic suppression at 12 months. A subset of participants will be interviewed at 12 months to learn about their HIV care experiences and, for those in the intervention arm, their experiences with the mLAR intervention. This protocol was developed in collaboration with the Baltimore City Health Department (BCHD) and the Maryland Department of Health (MDH) and with input from a community advisory board.

**Results:**

Enrollment began on February 25, 2020. As of August 11, 2022, 411 of the 450 target participants had been enrolled.

**Conclusions:**

Pragmatic implementation science trials designed with input from key stakeholders, including health departments and community members, can help evaluate the evidence for mHealth interventions to reduce HIV health disparities.

**Trial Registration:**

ClinicalTrials.gov NCT03934437; https://clinicaltrials.gov/ct2/show/NCT03934437

**International Registered Report Identifier (IRRID):**

DERR1-10.2196/42691

## Introduction

Advances in HIV treatment have markedly improved clinical outcomes among people living with HIV [1]. The United States has an ambitious strategy to end the HIV epidemic by 2030, but persistent racial and ethnic disparities in HIV outcomes must be addressed to achieve this goal [2]. African American people represent 13% of the population but account for 42% of people living with HIV and are less likely to receive antiretroviral therapy (ART) or achieve virologic suppression compared to White people [3]. Latinx people have higher HIV rates than non-Hispanic White people and are at high risk for delays in diagnosis and linkage to care [3,4]. Delayed initiation of ART is associated with increased morbidity and mortality, and high viral load is the main predictor of HIV transmission [5-8].

Modeling studies suggest that reengaging people living with HIV into care and suppressing their viral load is one of the most important interventions to reduce HIV incidence in the United States [9,10]. HIV retention in care and virologic suppression rates above 80% are needed to sharply reduce HIV transmission, but in 2021, only 66% of people living with HIV in the United States were suppressed [3]. HIV clinics have implemented various strategies to improve retention in care and adherence to treatment, such as automatic reminder calls for upcoming appointments, intensive case management, or systematic reviews of patients to identify those who have fallen out of care [11]. Evidence suggests that increased contact with the clinic can improve retention in care but may not be enough for people with substance use disorders and other unmet needs [12]. Several personal and structural barriers to care have been identified, including difficulty with transportation to the clinic, lack of information and practical support in navigating the system, and cost [13,14].

HIV retention and adherence strategies could be enhanced with the use of evidence-based mobile health (mHealth) strategies that leverage mobile phones to improve health outcomes. Systematic reviews have found that mHealth interventions can promote engagement in care, adherence to ART, and virologic suppression [15-17]. These platforms can be programmed to send tailored messages, reminders, and alerts to patients and providers using algorithms that leverage existing data. A distinct potential advantage of mHealth platforms is the ability to securely facilitate patient-centered approaches to retention in care efficiently and without overburdening providers, with alerts for action designed to focus on areas that truly require provider input. Studies from Baltimore demonstrate the feasibility and acceptability of using mHealth approaches with diverse populations, including African American men who have sex with men, people who inject drugs, and foreign-born Latinx people [18-20]. However, additional evidence is needed on the relative benefit of mHealth interventions in real-world settings to justify routine investments in mHealth resources and technology.

This manuscript describes the Leveraging mHealth and Patient Supporters for African Americans and Latinxs in HIV Care (LEAN) study protocol for a pragmatic type II hybrid effectiveness-implementation randomized controlled trial. This trial will compare existing standard-of-care clinic HIV linkage, adherence, and retention (LAR) protocols to an mHealth-enhanced linkage, adherence, and retention (mLAR) intervention with the hypothesized finding of improved virologic suppression and engagement in care in the mLAR study arm. This protocol was developed in collaboration with the Baltimore City Health Department (BCHD) and the Maryland Department of Health (MDH) and with input from a community advisory board. The study will evaluate the effectiveness of an mHealth intervention integrated into clinical and public health operations and examine implementation mechanisms and outcomes to inform broader scalability and sustainability.

## Methods

### Study Setting

This is a collaborative study between the BCHD, MDH, and Johns Hopkins University conducted in Baltimore City. The HIV prevalence rate in Baltimore is among the highest in US metropolitan areas, and it is a city that faces profound health disparities. In Baltimore, African American people have an HIV prevalence that is 5 times higher than among White people and account for more than 80% of all HIV cases; Latinx people have a higher prevalence of HIV than White people and are at the highest risk for late HIV diagnosis among all racial and ethnic groups [21].

### Study Design

**Figure 1:**
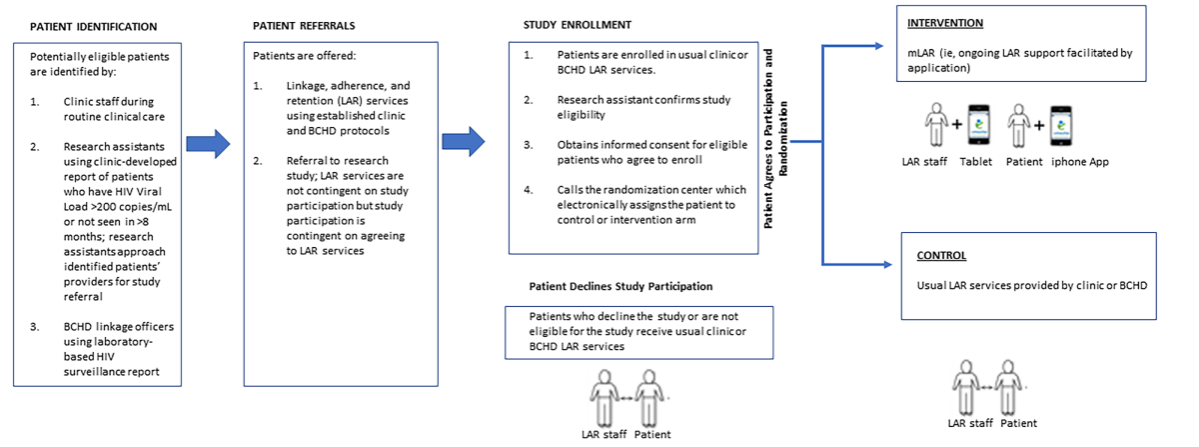
LEAN (Leveraging mHealth and Patient Supporters for African Americans and Latinxs in HIV Care) study design. BCHD: Baltimore City Health Department; LAR: linkage, adherence, and retention; mLAR: mobile health–enhanced linkage, adherence, and retention.

This study is a pragmatic type II hybrid effectiveness-implementation [22,23] randomized trial comparing standard LAR services to mLAR services (Figure 1). The study was conceptualized through discussions with patients, providers, linkage officers, and health officials regarding barriers and facilitators to engagement in HIV care and ways to leverage technology into existing services and systems to facilitate provider-patient communication. Study activities were reviewed and approved by the Institutional Review Board at the Johns Hopkins School of Medicine (IRB00195120). The study was registered at ClinicalTrials.gov (NCT03934437).

#### Conceptual Framework

**Figure 2:**
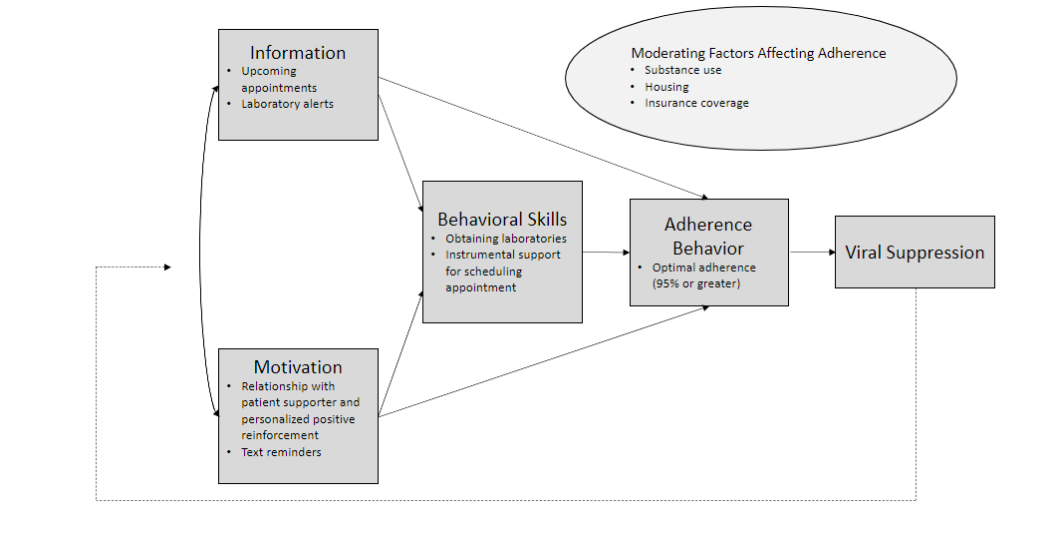
The situated Information-Motivation-Behavioral Skills (sIMB) Model of Adherence conceptual framework.

The mLAR intervention was informed by the situated Information-Motivation-Behavioral Skills (IMB) Model of Adherence, an evidence-based and theory-driven approach to understanding ART adherence and engagement in care (Figure 2). The situated IMB model focuses comprehensively on the information, motivation, and behavioral skills factors that are conceptually and empirically linked to ART adherence and specifies individual and social factors that may moderate the relationship between the IMB model constructs and ART adherence. At its most general level, the IMB model asserts that someone who is informed about medication adherence (ie, appointment reminders and lab alerts), is motivated to act, and has the behavioral skills necessary to act effectively (ie, objective and perceived abilities, facilitated by patient supporters) is more likely to adhere to ART over time [24,25]. Social determinants of health, such as housing, and individual factors, such as depression and substance use, moderate adherence and can create barriers to gaining information, being motivated, and acquiring or adhering to ART. Figure 2 highlights examples of specific components of how the mLAR intervention provides information, motivation, and behavioral skills to promote ART adherence. Patient supporters try to address moderating factors, such as depression and substance use, by facilitating referrals to appropriate resources and maintaining close communication with the patient’s case manager, as needed.

#### Formative Work and Community Engagement

Extensive discussions with stakeholders informed the study’s implementation and app design. The BCHD and MHD expressed a strong preference for a pragmatic “real life” study so that results could be generalizable to public health practice. A steering committee of old and new partners included African American and Latinx individuals affected by HIV, HIV clinicians, clinic-based staff (ie, patient supporters), BCHD linkage team leaders, public health officials, and members of the nonprofit sector. The SC met monthly during the planning phase and then quarterly once data collection started. The SC provides guidance on all patient-facing aspects of the study, including app characteristics and use (ie, app name, app logo, materials to assist participants with using the app), participant referral and recruitment processes, study closeout for participants, and the in-depth interview guide. Ongoing SC meetings provide an opportunity to discuss challenges and brainstorm solutions. The app development team included JH researchers, members of the health department, and emocha Mobile Health software engineers who helped develop a Health Insurance Portability and Accountability Act (HIPAA)–compliant mHealth app to facilitate engagement in care. Adaptations to the app were also made in response to feedback from the community advisory board and in-depth interviews with participants and patient supporters. Based on community advisory board feedback, the app was named NxtSTEP.

### Study Participants

#### Study Enrollment Criteria

Enrollment criteria include (1) being ≥18 years of age, (2) having the ability to provide consent, (3) having a new HIV diagnosis or being HIV-positive and “out of care” or being deemed by clinic staff as someone who could benefit from LAR services, and (4) living in Baltimore (within BCHD and Baltimore clinics’ catchment areas).

#### Participating Clinic and Program Selection Criteria

Criteria for LAR program participation are (1) BCHD or other Baltimore-based HIV program, (2) has an existing HIV-focused LAR program, (3) has existing patient supporters as part of the LAR program, (4) does not use any formal mHealth component for patient supporters to communicate with patients (beyond basic telephone calls and texting), and (5) the leadership and staff of the LAR program must be willing to participate.

#### Recruitment

Study participants are recruited from participating programs in 1 of 3 ways (Figure 1): (1) participating clinics identify newly diagnosed patients with HIV, those who are out of care and those who clinic staff determine could benefit from LAR services, offer LAR services to these patients, and refer interested patients to the study; (2) research assistants use clinic-developed reports to identify patients who could benefit from LAR services and approach these patients’ care providers during their regularly scheduled clinic visits or during hospitalization to refer interested individuals to the study; and (3) BCHD linkage officers use a laboratory-based HIV surveillance report from the state’s enhanced HIV/AIDS Reporting System (eHARS) that identifies newly diagnosed patients with HIV or those who are out of care, and when these patients are offered linkage services, the linkage officer offers study referral.

LAR services are provided regardless of study interest, but individuals who would like to enroll must agree to LAR support. Individuals interested in study enrollment are referred to a research assistant, who confirms eligibility, describes study participant responsibilities, and obtains informed consent.

#### Randomization

After obtaining consent, the research assistant enters the participant’s information into a Research Electronic Data Capture (REDCap) database [26,27], which has been programmed to randomly assign the patient to the control or intervention arm in a 1:1 ratio. The randomization schedule was generated by the study statistician, uses permuted blocks of varying sizes, and is stratified by whether the patient identified as Black or African American to ensure equal distribution of race in each study arm. Due to practical considerations, participants, clinic staff, and the study team are not blinded to arm assignment. Data analysis related to HIV clinical outcomes will be conducted in a blinded fashion.

#### Study Assessments

This is a pragmatic implementation trial with outcomes (eg, retention in care) that could be influenced by frequent study-related interactions, so study contact is minimized to a baseline visit where the research assistant obtains basic demographic and contact information and the reasons for referral to LAR services (ie, newly diagnosed, previously diagnosed but out of care, etc). A brief client satisfaction survey is conducted at the end of the 12-month follow-up period. Participants who agree to be contacted for an in-depth interview are randomly selected, aiming to capture both male and female participants in the control and intervention arms, including participants who did not use the app.

#### Participant Remuneration

All participants receive a US $50 incentive at initial enrollment in the study. Participants also receive US $20 for the completion of a client satisfaction survey and US $50 if they participate in an in-depth interview.

### Study Arms

#### Overview

Patients are randomized to the control arm or the intervention arm. Participants randomized to the control arm will receive the usual LAR services as per clinic. Participants randomized to the intervention arm will download the NxtStep app to their personal phones (or the study phone if the participant does not own a compatible phone) and be onboarded for using the app with the research assistant. Subsequently, the intervention arm participants will use the app to communicate with their patient supporter at the clinic or BCHD and receive algorithm-driven alerts (Table 1).

#### Standard LAR Services

Below are descriptions of the standard LTCR services at each site.

#### BCHD Linkage to Care Program

BCHD has patient supporters called linkage officers who attempt to contact and provide linkage services to all Baltimore residents newly diagnosed with HIV or who are presumed to be out of care [28]. These patients are identified using the state’s eHARS, which includes results for all HIV tests (including HIV viral load) conducted in Maryland, DC, and Virginia. An MDH algorithm generates weekly reports for the BCHD linkage team of new HIV diagnoses or out-of-care patients in Baltimore (defined as patients with a viral load >50,000 or no HIV labs for >12 months). The linkage officers attempt to contact patients on this list, and those who agree to be linked to HIV care by linkage officers are provided a same-day appointment and a warm handoff to clinical care (ie, the outreach worker drives the patient to the clinic of his or her choice).

#### Clinic-Based LAR Programs

The Ryan White–funded HIV clinics currently participating in our study (Johns Hopkins Bartlett Clinic, BCHD Sexual Health Clinic, University of Maryland THRIVE Clinic, University of Maryland JACQUES Initiative) have patient supporters (called patient supporters, advocates, navigators, or health workers) who remind patients of upcoming appointments and act as a point of contact to help patients navigate their care. They help patients schedule appointments, lab visits, and medication refills. They also provide counseling and education to help patients self-advocate and connect to necessary services (eg, mental health services, substance use disorder treatment, or social services). Except for appointment reminders, which are intended to reach all patients, other services are delivered in response to patient or provider requests, not systematically to all patients. All clinics have rapid ART programs, and the patient supporters assist newly diagnosed patients through this process (quick appointment and introduction to the clinic). None of the programs provide home visits or services outside the clinic.

#### Intervention (mLAR)

This approach leverages existing LAR services at each study site and adds a novel mHealth component. The mHealth enhancement consists of 2 smartphone apps (NxtSTEP app), 1 for patients and 1 for existing patient supporters, to help facilitate communication on issues related to HIV care (eg, appointment scheduling, laboratory reminders, transportation), as well as patient-directed requests. The NxtSTEP app is designed to facilitate secure communication between the patient and patient supporters and to automate certain tasks, alerts, and reminders so that patient supporters can focus their efforts on patients at high risk of falling out of care (eg, calling a patient with a high viral load) rather than on time-consuming tasks not specifically targeted to high-risk patients (eg, general appointment reminders).

**Figure 3:**
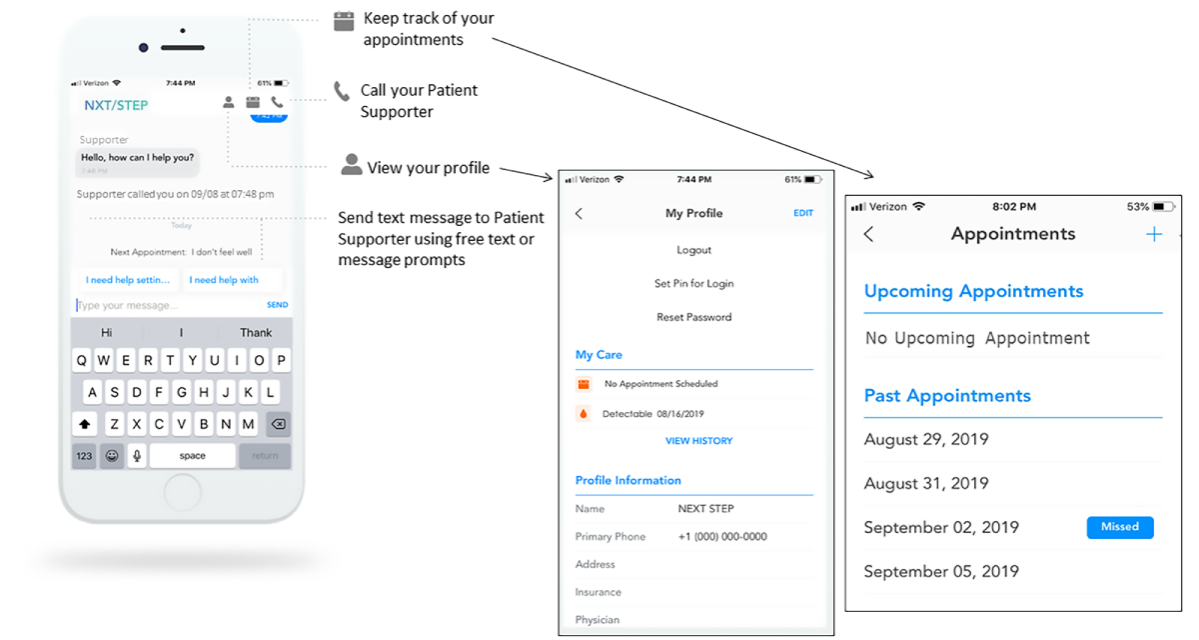
Patient view of NxtSTEP app.

**Figure 4:**
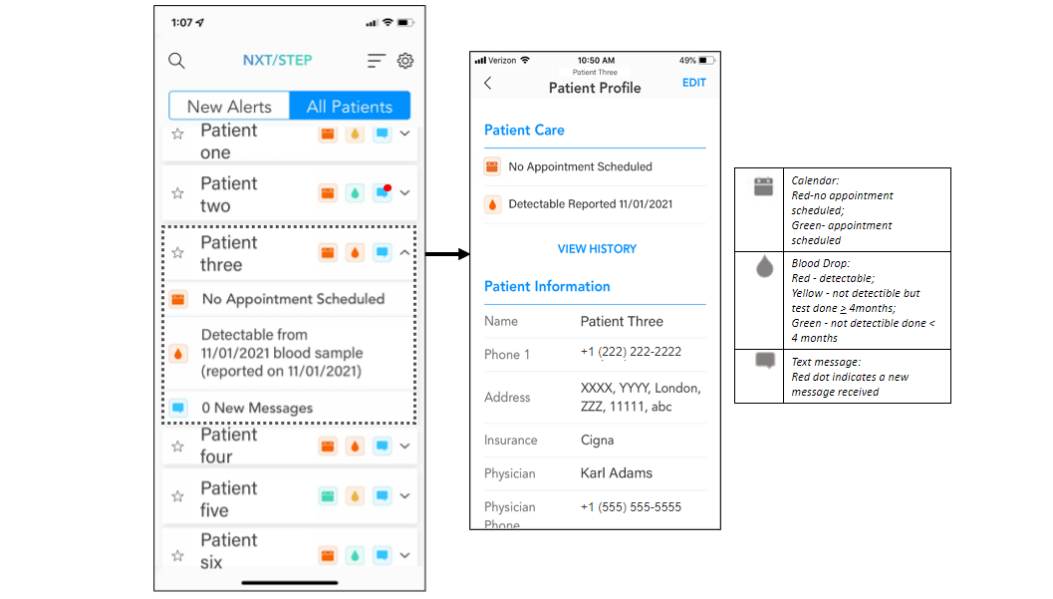
Patient supporter view of NxtSTEP app.

The NxtSTEP app has a viral load–driven algorithm using MDH HIV surveillance data that automatically alerts patient supporters and patients if the viral load is not suppressed (>200 copies/ml) or if more than 4 months have passed since the last viral load. This interval is consistent with HIV guidelines to monitor viral load at least every 3 to 4 months, or more frequently as clinically indicated, in patients who have not had sustained virologic suppression for more than 2 years [29]. In addition, patients are prompted to enter upcoming appointment dates in an app calendar, which allows for automatic reminders. Patient supporters are alerted if a patient does not have an upcoming appointment or if the patient missed their appointment. Patient supporters are trained to contact the patient alerted (lab alert, appointment alert, or patient message alert). Figures 3 and 4 show the patient and patient supporter’s NxtSTEP views, respectively. Tasks, alerts, and action prompts are outlined in Table 1 for the patient app and in Table 2 for the patient supporter app.

**Table 1:** Patient NxtSTEP app.

Task	Description	Patient support staff action
Appointment data	Tab to record the date, time, and place of an upcoming HIV care appointment	Patient support staff or patient can enter appointment data
Appointment reminders	Patient automatically receives reminders about upcoming appointments (3 days prior). The day after the scheduled appointment, patient receives the prompt, “Did you go to your appointment yesterday?” (Yes or No) If “Yes,” patient received a message saying, “Great job keeping your appointments!”	Patient support staff contacts patient, if alerted (see Table 2)
Ability to directly message patient support staff	Patient can click on prompts (eg, “I need help setting an appointment” or “I need help with”) or send a free text message to patient support staff	Patient support staff contacts patient, if alerted (see Table 2)
Control over messages	Patient has the option to type STOP if he or she prefers not to receive a type of message	Not applicable

**Table 2:** Patient support staff NxtSTEP app.

Task	Description	Patient support staff action
HIV data of participants in mLAR^a^ arm	Participant viral load (dated) automatically uploaded from eHARS^b^ (through BCHD^c^ lab-based reporting surveillance protocols)	Value not applicable
Viral load status alerts	Automatic “out of care” alert if no viral load for >4 months or viral load >200	Patient support staff communicates with the participant. Contact attempts are recorded.
Automatic appointment alerts	Automatic alert if no next appointment is documented in the patient app. “Missed appointment” alert if patient responds ‘No’ or does not respond to prompt, “Did you go to your appointment yesterday?”	Patient support staff communicates with participant. Contact attempts are recorded.
Patient initiated direct messaging	Automatic alerts for all patient-initiated messages, for example, “Patient needs help with appointment,” “Patient needs help with transportation,” or if the patient has typed a request with free text.	Patient support staff communicates with participant. Contact attempts and outcomes are recorded. For social determinants (housing, transportation, and substance use), patient support staff assists patient with access to community and clinical-based resources.

^a^mLAR: mobile health–enhanced linkage, adherence, and retention.

^b^eHARS: enhanced HIV/AIDS Reporting System.

^c^BCHD: Baltimore City Health Department.

NxtSTEP is available in English and Spanish. Spanish-speaking patients with limited English proficiency (LEP) are assigned a bilingual patient supporter who communicates with them in their preferred language. The study team had experience with cultural and linguistic adaptations of interventions and elicited feedback from the steering committee (see below) to ensure messages were tailored to the health literacy and language proficiency of the target population [30,31].

### Outcomes

#### Clinical Outcomes

The primary outcome of the trial is virologic suppression at 12 months, defined as a viral load <200 copies/ml within a 6-month window (ie, 3 months before or after the 12-month follow-up). The secondary effectiveness outcome of the trial is retention in care at 12 months, defined as having a viral load within a 6-month window (ie, 3 months before or after the 12-month follow-up). Data for the primary and secondary outcomes will be obtained from the MDH surveillance HIV viral load data reported to eHARS. All laboratory assessments will be conducted as part of clinical care. Participants with a suppressed viral load (HIV RNA <200 copies/ml) within the follow-up period will be fully engaged in HIV care. While some individuals can maintain immune control over HIV in the absence of ART, this occurs in only <1% of infected persons [32].

#### Implementation Outcomes

Using a hybrid study design, we incorporated implementation science evaluation methods to assess important implementation questions [33]. Hybrid designs are efficient approaches to obtaining information that can help understand research findings in terms of internal validity and external generalizability and provide data to facilitate translating research into real-world policies and programs [34]. LEAN implementation processes will be evaluated in terms of core factors in the Reach, Effectiveness, Adoption, Implementation, and Maintenance (RE-AIM) approach (Table 3) [33] and informed by concepts from the IMB theoretical framework.

IMB framework dynamics will be explored through open-ended qualitative methods. The evaluation will engage the intervention’s consumers (HIV-positive patients randomized to mLAR) as well as other stakeholder groups, including patient supporters, clinicians, and public health officials at the BCHD. Engaging this range of stakeholders will provide a holistic understanding of the barriers and facilitators to successfully implement the intervention, enhance service delivery, and assess the feasibility of scaling up the intervention if it is effective.

**Table 3:** Summary of mobile health–enhanced linkage, adherence, and retention implementation evaluation using the Reach, Effectiveness, Adoption, Implementation, and Maintenance approach.

Component	Research question	Assessment methods	Outcomes of interest
Reach	Who received the intervention?	Program data In-depth interviews	Proportion of eligible patients successfully engaged in intervention. Differences between those reached and those not reached. Reasons why.
Effectiveness	What impact did the intervention have on patient-oriented outcomes?	End-of-study viral load In depth interviews	Viral suppression Engagement in care Acceptability of the intervention
Adoption	Did the providers, patient supporters, and staff (implementers) adopt the intervention?	Program data, in-depth interviews	Frequency of contacts between patient supporters and patients. Services provided and received. Perceptions and attitudes of implementers.
Implementation	Was the intervention delivered as intended?	Program data, in-depth interviews	Program fidelity. Adaptation to design over time.
Maintenance	Was the intervention sustained and institutionalized over time?	Program data, in-depth interviews	Consistent implementation and effectiveness over time. Retention.

#### Client Satisfaction Survey

All participants (control and intervention, n=450) will be asked to complete a brief client satisfaction survey using a validated Client Satisfaction Questionnaire (CSQ-8) [35]. Participants in the intervention arm will be asked to complete the validated System Usability Scale to assess the acceptability and ease of use of the NxtSTEP app [36].

#### Qualitative Data

In-depth interviews will be conducted with 3 key stakeholder groups: patient supporters, study participants, and clinic leadership and public health officials. Interviews with patient supporters will occur before and 12 months after the implementation of mLAR to assess the nature of service delivery, client interactions and how this differs from standard linkage activities, barriers and facilitators of engaging out-of-care patients, challenges and solutions to the service delivery, and recommendations for service delivery improvements and expansion of reach. We will also conduct interviews with study participants randomized to the control or intervention arms. All participants will be asked about their experiences living with and engaging in HIV care, including facilitators and barriers to appointments and medication adherence, past experiences dropping out of care, impressions of HIV-related services they received, as well as recommendations for additional services. Participants randomized to the intervention arm will also be asked about their experience using the app, their satisfaction with features of the app such as the automated messaging they received, their motivation for engaging the patient supporter–delivered SMS text messages, their perception of the impact of app usage on their care, and their suggestions for other ways the mobile phone may facilitate HIV retention in care and adherence to medications. We will also assess facilitators and barriers to app use. Interviews with clinic leaders and public health officials will explore perceptions of the intervention’s integration into linkage and engagement of care activities at clinics or the health department, as well as recommendations for service delivery improvements. These interviews will provide insights about policy, acceptability, political buy-in for the intervention, and the potential for sustainability and adoption in other settings going forward.

#### Process for Feedback and Intervention Refinement

During implementation, results from the interviews and client satisfaction surveys will be shared at regular intervals with patient supporters, program administrators at the BCHD, and the steering committee. Sharing these data will facilitate the use of the information for problem-solving, community engagement, and an opportunity to improve the quality of the intervention.

### Statistical Procedures

We will conduct a test for the equality of proportions of virologically suppressed, not suppressed, or missing viral load individuals at 12 months between the 2 study arms, in line with the randomized design of the study. Secondary to this, and to adjust for any residual confounding, we will conduct a log-binomial regression using individual outcomes (yes or no suppressed) with the study arm as the main predictor and adjusting for patient demographic characteristics, socioeconomic status at baseline, and other factors. The observed virologic suppression in the control arm is higher than the estimate used in the design phase of the study. We will thus further conduct a difference-in-differences analysis to evaluate the effect of the study arm over time. This will be conducted using a log-binomial regression and will investigate the time (enrollment vs 12 months) by arm interaction term, adjusting for baseline sociodemographic covariates and robust standard errors to account for within-participant clustering.

A subgroup analysis will be conducted to assess the effects of the intervention among African American people and people of Hispanic or Latinx descent, and if most of our recruited participants come from 1 site, we will conduct a subgroup analysis focusing on this site alone. Sensitivity analysis using propensity-score weighting to adjust for imbalances in study arm allocation by site will be conducted.

### Power Calculation

The reported virologic suppression or lack of a viral load measurement rate under standard of care among HIV-positive African American and Latinx people in Baltimore City is 33% [37]. In order to detect an improvement of 13%, such that 46% of the mLAR group have a viral load and achieve virologic suppression with 80% power at an *α*=5% level of significance, a total sample size of 442 (n=221 per arm) is required. A total of 450 participants will be enrolled in the study to account for participants who decide to withdraw from the study after enrollment. Because this is a pragmatic implementation study and the primary outcome is viral load obtained from surveillance data, patients who are lost to follow-up will still be included in the analysis. According to the protocol, patients who are lost to follow-up and do not have a viral load recorded in the surveillance database within the window period will be considered virologic failures.

### Qualitative Data Analysis

Transcripts from in-depth interviews and key informant interviews will be entered and managed in Atlas.ti (Scientific Software Development GmbH). Separate coding schemes will be developed for HIV-positive patients (the control and intervention arms) and the other staff (patient supporters, program managers, and health officials). An iterative coding process will be used to conceptually name the data and reduce it to manageable units of information that cover broad and general categories. Codes will be informed by the questions in the qualitative guides, and new themes that emerge from the data will be analyzed through a constant comparison approach, allowing for themes to emerge and ensuring that the knowledge assembled from the observational data is not subjected to the ideas solely established through the interview guide. For each group, 2 coders will conduct open-coding on 3 transcripts to develop an initial coding scheme. The draft codebook will continue to be revised as needed as additional transcripts are coded and discussed. Once a final coding scheme is established, the transcripts will be systematically coded. Through weekly meetings, a team approach to data analysis will be used, whereby different analysts provide feedback on emerging interpretations and check emerging categories against the raw data. In this way, an “audit trail” will be used to help ensure the trustworthiness of findings, gather input from multiple perspectives, and enhance reliability.

### Data Management and Quality Assurance

Data collected by the NxtSTEP app is sent in real time to a secure server, where linkage activities and data can be accessed through a simple web interface. Only authorized study personnel have access to this data. A triple level of data encryption is used to ensure confidentiality of data and HIPAA-compliance. Patient supporter devices and access to the patient supporter and patient apps are password protected. To further protect patients’ confidentiality, the patient’s app and messages to the patient include nonspecific terms without mention of HIV or other personal health information. Data will be cleaned at Johns Hopkins and deidentified before analysis.

### Ethics Approval

This study was approved by the Johns Hopkins School of Medicine Institutional Review Board (IRB00195120).

## Results

Study enrollment began February 2020 and was suspended in March 2020 until June 2020 due to the COVID-19 pandemic. As of August 11, 2022, 411 people had been enrolled in the study. Study enrollment of 450 participants was completed on December 1, 2022. Follow-up of these participants will continue until early 2024, and study assessment and data collection will be completed by January 31, 2024.

## Discussion

This trial should provide evidence on whether an mLAR intervention improved viral suppression and engagement in care when compared to existing standard-of-care clinic HIV LAR protocols. Compared to prior work, this study will generate rigorous randomized, controlled trial-level evidence with hard biologic outcomes for an intervention used within real-world programs. Study strengths include the rigorous study design, multiple study sites, and a user- and community-informed app design and implementation. Study limitations include potentially imbalanced recruitment from the various study sites, COVID-19–related adaptations, and unforeseen technological challenges.

Informed by the community advisory board, we will implement (or develop) a robust dissemination plan that includes not only peer-reviewed publications and presentations at scientific conferences but also presentations among community-based organizations that offer HIV services and to city and state policy makers and funders of HIV programs.

The rigorous evaluation of a pragmatic HIV LAR to care approaches, especially in settings like Baltimore, where African American and Latinx people carry a disproportionate burden of HIV infections, is desperately needed. The results of this randomized study will contribute to the evidence on the effectiveness of an mHealth-enhanced LAR initiative implemented by the local health department and clinics that provide care to people living with HIV. Evidence supporting the feasibility, effectiveness, and implementation considerations of this intervention will be important for the BCHD and other health departments as they make decisions on the allocation of limited resources to address the HIV epidemic in their jurisdictions. As value-based reimbursement becomes the norm for payors including Medicare and Medicaid, this study will contribute to the evidence regarding insurance coverage for mHealth interventions to improve patient outcomes. The information will also be relevant to Ryan White officials responsible for allocating federal dollars to support clinical and ancillary services to improve HIV outcomes, particularly among patients who are uninsured or underinsured. Finally, and most importantly for patients and providers, the primary outcome of this study (virologic suppression) is directly related to improved clinical outcomes (improved survival and lower morbidity) and reduced HIV transmission in affected communities.

In summary, the LEAN trial seeks to test the impact of an mHealth-enhanced linkage to care, adherence, and retention strategies among vulnerable, mostly minority persons living with HIV in a real-world setting. If found to be effective, potential future directions for this intervention include scaling it up and adapting it to other settings as an important tool to reach HIV epidemic control.

## Appendix

Multimedia Appendix 1 Peer-review report from Patient-Centered Outcomes Research Institute (PCORI).

## Abbreviations

ART: antiretroviral therapy
BCHD: Baltimore City Health Department
eHARS: enhanced HIV/AIDS Reporting System
HIPAA: Health Insurance Portability and Accountability Act
IMB: Information-Motivation-Behavioral Skills
LAR: linkage, adherence, and retention
MDH: Maryland Department of Health
mHealth: mobile health
mLAR: mobile health–enhanced linkage, adherence, and retention

## Data Availability

After completion of this study and publication of primary and secondary trial outcomes, a deidentified data set will be submitted for publication to JMIR Data.
