# Supplementary material for: Leveraging mHealth and Patient Supporters for African Americans' and Latinxs' Engagement in HIV Care (LEAN): Protocol for a Randomized, Controlled, Effectiveness-Implementation Trial
Source: JMIR Res Protoc. 2023 Feb 14;12:e42691. doi: 10.2196/42691 (PMC9975915; doi:10.2196/42691)
Supplement: Multimedia Appendix 1 [file resprot_v12i1e42691_app1.pdf]

PATIENT-CENTERED OUTCOMES RESEARCH INSTITUTE
SUMMARY STATEMENT
(Privileged Communication)

Principal Investigator: Kathleen Page

Organization: Johns Hopkins School of Medicine

Project Title: Leveraging mHealth and peers to Engage African-Americans and Latinos in HIV care (LEAN)

PCORI Funding Announcement: Addressing Disparities

Review Cycle: Cycle 1 2018

Request ID: 11244

AVERAGE OVERALL SCORE: 27

QUARTILE: 1

In-Person Review Discussion Notes:
Strengths:

- This application is focused on addressing an important health disparity in HIV care. The application clearly identifies a critical gap in the literature on how best to reach African-American and Latino HIV-infected individuals who do not have regular linkages to healthcare.
- This study supports a current demand for evidence-based mobile health strategies for HIV-infected African American and Latino patients.
- The proposed mHealth enhanced linkage-to-care and retention intervention would help facilitate patient-directed and treatment-focused communications linkage officers. Building a longer-term relationship between patient and linkage officer would fill a current gap in care.
- The application presents a clear conceptual framework that guides the intervention.
- The decision to recruit patients from a community setting is a strength. The evaluation of the proposed intervention in this real-world public health setting can help inform decisions to adopt this intervention in other public health departments.
- The research team is well experienced with conducting research in this target population. Johns Hopkins University and the Baltimore Department of Health have a strong partnership.
- This study is patient-centered and practical for an urban health setting.
- The application demonstrates strong engagement with the Baltimore City Health Department and other key stakeholders.

Weaknesses:

- Some reviewers were concerned that the ability to detect differences in the primary outcome (collected using surveillance data) could be hampered by missing data in the non-mHealth usual care arm.

- Some reviewers noted that the cost of the mHealth intervention might limit future implementation and scalability. This was considered a moderate weakness.
- The anticipated 10% drop-out rate seems low for this target population. Sufficient justification should be provided.
- The steering committee
  - Lacks representation from payers which could help provide insight on the sustainability of this intervention.
  - Is proposed to meet on a quarterly basis once the study begins, which was considered too infrequent by a few reviewers. This was considered a minor weakness.

The following reviewer critiques were completed prior to the in-person review and were not altered post-discussion.

Criterion 1: Potential for the study to fill critical gaps in evidence

Reviewer 1:

Strengths:

- Determining whether a Baltimore City Health Department (BCHD) mHealth-enhanced Linkage to Care and Retention (mLTCR) intervention can improve HIV outcomes among HIV-infected African American and Latinos could address these deficiencies in the state of the science.
- This is especially the case as these individuals in Baltimore have no regular links to health care.

Weaknesses:

- None noted.

Reviewer 2:

Strengths:

Weaknesses:

Reviewer 3:

Strengths:

- The application does a good job of elucidating the critical need and gap in current knowledge regarding health outcomes disparities in African Americans and Latinos with HIV, citing multiple previous research prioritizations, who are less likely to receive antiretroviral therapy (ART) or achieve virologic suppression compared to whites.
- The applicants have clearly described the challenges faced by providers in re-engaging patients in care due to lack of linkage officials' ongoing relationships with patients and contact information obtained from often out of date surveillance databases pointedly indicating that current clinic-based retention in care initiatives have proven insufficient to retain individuals out of care who may be homeless, transitionally housed, transient,

and/or without working phone numbers.

- The applicant has clearly delineated the urgency to tailor approaches based clients' particular needs and circumstances, including longitudinal retention support during their HIV care trajectory to facilitate future re-engagement attempts, if needed.
- Providing linkage and longitudinal retention (LTCR) services from the health department that complements existing clinic-based retention efforts has not been systematically evaluated; the research findings from this study has the potential to fill these evidence gaps.

Weaknesses:

- (Minor weakness) mHealth interventions can promote engagement in care, adherence to ART, and virologic suppression using algorithms that leverage existing surveillance data and patient input. The application includes discussion of current variations in practice patterns regarding mHealth applications to facilitate engagement in care and to link patients to services and improve coordination of care by public health departments for tuberculosis, hepatitis C, and opioid use disorder treatment. However, the applicants do not report the efficacy of these other previously reported programs (just the feasibility and acceptability of the mHealth implementation of the cited studies).

Reviewer 4:

Strengths:

Weaknesses:

Reviewer 5:

Strengths:

- In Baltimore, African Americans have five times the rate of HIV infection compared to whites, and account for 78% of all new infections. Improvement in linkage and retention in care are indicators of most importance to reduce HIV in the U.S. Rigorous evaluation of linkage and retention strategies are needed.
- Health Department-driven linkage strategies using HIV surveillance data have led to some success but have limitations. Retention to care protocols in clinical settings are heterogeneous, depending on funding streams, needs of patients served, and leadership priorities.
- Evidence that mHealth interventions can improve HIV outcomes by facilitating patient-provider communication that provides information, motivation, and behavioral skills (IMB model) to improve ART adherence. Additional evidence needed on the relative benefit of mHealth interventions in real-world settings to justify routine investments in mHealth resources and technology.
- The study will evaluate whether a health department-driven mHealth-enhanced Linkage to Care and Retention (mLTCR) intervention can improve HIV outcomes among HIV-infected African Americans and Latinos compared to standard Baltimore City Health Department linkage to care protocols. Findings have the potential to fill important gaps in the evidence-base for linkage to care interventions. Results will contribute to the evidence on the effectiveness of mHealth-enhanced LTCR initiatives implemented by the local health department.
- Feasibility and effectiveness of this intervention will be important for the BCHD and other health departments as they allocate limited resources to address the HIV epidemic in their jurisdictions. The information will also be relevant to Ryan White officials responsible for allocating funds.
- Outcomes of this study (virologic suppression, linkage and retention in care) are directly related to improved clinical outcomes (improved survival and lower morbidity) and reduced HIV transmission
- Proposed research would fill critical gaps identified. Improvement in linkage and retention in care are indicators of most importance to reduce HIV in the U.S. Rigorous evaluation of linkage and retention strategies are needed.

Weaknesses:

- None noted.

Criterion 2: Potential for the study findings to be adopted into clinical practice and improve delivery of care

Reviewer 1:

Strengths:

- The research team will employ an iterative process of dissemination throughout the project period, disseminating findings to all partners and stakeholders, including study participants, through multiple venues and in ways that are “understandable, respectful, and useful”.
- A dissemination subcommittee at the end of year 2 to coordinate dissemination outreach to participants and other stakeholders. This subcommittee will utilize the PCORI Dissemination and Implementation Toolkit worksheet.
- The intervention platform will be available at a reduced cost for the BCHD and other health departments to ensure long-term sustainability if the intervention is effective.
- In all this is a well thought-through dissemination plan with excellent detail for local dissemination.

Weaknesses:

- Fewer details are provided for broader national dissemination than for local work.
- The expensiveness of this approach even if effective could preclude broad dissemination. This is reflected in a variety of places, especially the requested $6.4 million budget.

Reviewer 2:

Strengths:

- This application supports the demand for evidence-based mobile health strategies that can significantly promote patient engagement in care and improve health outcomes such as adherence to ART and virologic suppression for African-American and Latino HIV infected patients. This is a MODERATE STRENGTH.
- This study uniquely aims to address the overwhelming challenges often faced by HIV infected urban minority populations (poverty, substance use disorder and housing instability) by putting patients in direct phone app two-way communications with linkage officers (outreach workers) who can help coordinate needed care and timely treatment over a 12-month period. This is a MAJOR STRENGTH.
- Research findings from this study are directly related to improving clinical outcomes in virologic suppression, improved survival and lower morbidity rates of HIV infected minority patients and reduce HIV transmission in other affected communities. This is a MAJOR STRENGTH.

Weaknesses:

- Since this study does not have a multi-site, regional or state HIV-infected target patient test population, research findings may not be readily accepted or adopted as health policy. This is a MINOR WEAKNESS.

Reviewer 3:

Strengths:

- The decision to recruit patients from the community setting (e.g., public health department) and not a clinic only setting strengthens the ability of the findings to be generalized to the general patient population.
- The application describes a pragmatic study that can inform decision-makers as the protocol will be replicable in other public health settings similar to the Baltimore County Health Department (BCHD). It is likely if the intervention is effective it could be reproduced by other county health departments to promote better patient outcomes.
- (Major strength) The use of smartphones has become ubiquitous among nearly all patient populations. The approach that targets HIV linkage and retention in care using technological innovation, i.e., smartphone algorithms, is highly innovative, has the potential to improve healthcare delivery in a meaningful way.
- The study's outcomes (viral load and retention in care measured from surveillance data) are very relevant to public health institutions and funding agencies like Ryan White and can be evaluated in other public health settings as part of routine HIV surveillance.
- The application provides information that supports demand for this kind of a study from end-users. The application describes input from stakeholders/patients regarding the need for a less homogenous one size fits all intervention and a more tailored, patient-centered interactive intervention such as the one proposed in this study.

Weaknesses:

- (Minor weakness) A potential barrier that could hinder adoption of the intervention by others is the lack of community-based linkage officers and/or privacy issues outside of large urban areas (e.g., small towns, rural settings) as well as cost, existing programming, and available resources.
- (Minor weakness) A minor weakness but of note is that the application describes a Steering Committee that will have a dissemination subcommittee to tailor the dissemination of findings to stakeholders using the PCORI Dissemination and Implementation Toolkit worksheet to guide messaging in order to facilitate implementation of the intervention in other settings, but does not go into any detail offer suggestions on how they might overcome any of challenges in dissemination and/or implementation in other settings.

Reviewer 4:

Strengths:

- This study is a collaborative study between Baltimore City Health Department (BCHD) and Johns Hopkins University, which utilizes an existing public health infrastructure. Evaluating the proposed intervention in this real-world public health setting can help to inform decisions to adopt this intervention at other public health departments if it is shown to be effective. Major strength.

Weaknesses:

- The dissemination plan will be implemented in year 3 of the project, when it will determine what research findings and products will be shared with participants and stakeholders, identify stakeholder group types, determine what partners can facilitate dissemination, develop channels of communication such as interactive presentations, develop a process evaluation plan to monitor dissemination activities, and a timeline for

dissemination. At this point, the dissemination plan lacks specificity. It would be preferable to start these activities earlier in the study, and to even give consideration to how the information will be disseminated prior to beginning the study. Moderate weakness.
- Cost is a barrier to implement this intervention in other settings if it is found to be effective. Having payors represented as key stakeholders and having a more robust dissemination plan for findings earlier in the process could help reduce this barrier. Moderate weakness.
- The application lacks specificity about their local and national stakeholders and their strategies to engage them. Major weakness.
- The application provides information that supports a demand for this kind of a study from providers, but evidence of a demand from patients is lacking. Moderate weakness.

Reviewer 5:

Strengths:

- This pragmatic trial will be implemented by the BCHD Linkage to Care program because it leverages an existing public health infrastructure to identify and reach out to HIV-infected individuals who are out of care throughout the city (regardless of which specific clinic they attend).
- Evaluating this intervention in a real-world public health setting will increase the generalizability of the results and has the potential to inform practice at health departments throughout the country.
- The patient application will be available in English and Spanish
- Spanish-speaking limited English proficiency patients will be assigned a bilingual linkage officer who will communicate with them in their preferred language.
- Long-term goal is to develop an effective linkage and retention to care intervention that improves HIV clinical outcomes, especially among minority populations, and can be realistically integrated into the workflow of health departments.
- The results of this randomized study will contribute to the evidence on the effectiveness of mHealth-enhanced LTCR initiatives implemented by the local health department. Evidence supporting the feasibility and effectiveness of this intervention will be important for the BCHD and other health departments as they allocate limited resources to address the HIV epidemic in their jurisdictions. The information will also be relevant to Ryan White officials responsible for allocating federal dollars to support clinical and ancillary services to improve HIV outcomes, particularly among patients who are uninsured or underinsured.

Weaknesses:

- None noted.

Criterion 3: Scientific merit (research design, analysis, and outcomes)

Reviewer 1:

Strengths:

- The scientific objective of comparing virologic suppression and retention in care between HIV-infected individuals who are out of care randomized to receive standard BCHD linkage services versus mLTCR services is well supported by the methodology.
- The primary outcome is viral load suppression (< 200 copies/ml) 12 months after study enrollment, and the secondary outcome being retention in care, both highly appropriate for this effort.
- Emocha Mobile Health is a pioneering mobile health company with experience in clinical trials, population health

management, and customized clinical applications with high-impact potential for research and real-world use with a full complement of expertise in mobile application development, including project management, software engineers, server/database architects, and user interface designers to ensure efficient care delivery and ease of use through clean, concise, digital design. They have the appropriate range of skills to provide an effective platform for this study.

- The argument for a “pragmatic” rather than a scientifically sampled and randomized trial is understood as the best way to reach these out-of-care patients.
- The investigators propose a well detailed examination of the implementation of mLTCR services using a mixed-methods approach among patients, local/state public health stakeholders, and medical providers to determine the intervention’s feasibility, acceptability, coverage, fidelity, sustainability, and patient satisfaction. This is highly detailed and fits well with the overall study.
- The recruitment, randomization, and quantitative as well as qualitative analysis approaches are highly detailed and appropriate.
- Power is adequate for testing the study hypothesis.
- Given the patient population this study will push the limits of ‘feasibility.’ However, this seems to be the right research partnership to give it a try.

Weaknesses:

- It is not clear whether power would be sufficient for ethnic, gender and age category subgroup analyses.
- The researchers note that the primary outcome measure (documented VL < 200) may underestimate virologic suppression since some patients who do not have a viral load within the time frame may be miscategorized. Although it is true that this problem may affect both arms of the study equally, the external validity of the evidence may be compromised.
- The ‘pragmatic study design’ may further limit external validity as the participants may have different availability for or reasons to participate in the trial.
- Participants randomized to the intervention arm will be asked if the text message topics and materials in the patient portal were important to them, their motivation for engaging the linkage officer-delivered text messages, and all participants will be given client satisfaction surveys. These seem highly subject to social desirability.

<u>Reviewer 2:</u>

Strengths:

Weaknesses:

<u>Reviewer 3:</u>

Strengths:

- (Major strength) The application describes the conceptual framework, IMB-skills model to inform the design, key variables, and relationship between interventions and outcomes being tested; it is appropriate for this study. The IMB-skills model has been used extensively in intervention studies and provides a framework of adherence-related barriers that generally fall within information, motivation, and behavioral skills areas, and can be used as a tool to quickly identify potential adherence related deficits in practical settings.
- (Major strength) The research plan adequately describes rigorous methods. The design of the proposed study proposed (a 2-arm pragmatic RCT) is justified, and the patient population (African Americans and Latinos with HIV) and study setting (e.g., public health department) are appropriate to answer the research question. Each of the comparators is described clearly and well-justified, and both will it be sufficiently measured.

- Sample sizes and power estimates are appropriate for this proposed RCT.
- The project timeline is feasible, given the target sample of 500 (250 in each arm) and plans to address patient or site attrition are adequate.

Weaknesses:

- (Moderate weakness) The application explains how the qualitative data (i.e., face to face interviews) will be analyzed but does not explain the plan and analysis for the proposed 'mixed methods' approach, such as timing (i.e., sequential or concurrent) of the quantitative and qualitative components or quality of the quantitative data. For example, if sequential, the first phase of data collection should help to inform the second phase, or the second phase is used to aid in the interpretation of data collected in the first phase. Another concern is the weight given to each component phase; it appears the 'mixed methods' relied heavily on qualitative data and therefore may not be considered a true mixed methods study. The applicant has not fully explicated if deductive logic will be used to weight the quantitative portion more heavily and seek to explain the phenomenon, or if inductive or exploratory logic will emphasize the qualitative portion. Also, the application needs to provide a more detailed discussion on how the qualitative data and quantitative data will be used (including which quantitative data will be used) for a mixed methods approach.
- (Minor) The application describes the use of a grounded theory approach, but no rationale is offered as to why this is chosen over other forms of qualitative analytic approaches.
- (Minor) The application does not provide the questions/items in the interview guide (e.g., one question, semi-structured or otherwise) for the qualitative interviews.
- (Minor) An essential component of the study relies on linkage officers engaging and linking clients to healthcare services, encouraging the use of these services and continued interaction via mHealth technology (i.e., smartphone). It is unsure how variations in linkage officers' abilities, experience, and training in this practice could affect study outcomes; proposal does not thoroughly discuss how those linkage officers will be trained and how/whether their interactions with patients will be standardized or monitored. Moreover, there is no mention of accounting for other confounders, such as a physician or other clinic staff influences in patient engagement.
- (Moderate) The application utilizes the IMB Skills Model of Adherence, which has been proven effective for persons living with HIV/AIDS, highly appropriate for a study that aims to inform, motivate and engage patients for positive change (e.g., adherence). Possible modifying factors affecting adherence (substance use, housing, insurance coverage) are included (yet not integrated) in the model. However, cultural, racial, and linguistic factors are insufficiently discussed as possible modifiers.
- (Minor weakness) This study seems ambitions without having pilot data directly related to the proposed mHealth intervention, aside from any initial pilot studies of the Emocha (technology) platform and reports of numbers of patients not linked to care.

<u>**Reviewer 4:**</u>

Strengths:

Weaknesses:

<u>**Reviewer 5:**</u>

Strengths:

- Robust conceptual framework, Information-Motivation-Behavioral Skills (IMB) model of adherence, an evidence-based and theory-driven approach (represented in Figure 1).
- A 4-year pragmatic individual randomized controlled trial comparing standard BCHD linkage services (control) to mHealth-enhanced LTCR services (intervention).

- The study will not be masked to participants or linkage officers due to practical considerations, but all data analysis related to Aim 1 will be conducted in a blinded fashion.
- Enrollment criteria will include 1) ≥ 18 years of age, 2) ability to provide consent, 3) Identified by BCHD Linkage protocol to be a new HIV diagnosis or HIV-infected and "out of care," and 4) live in Baltimore City (within BCHD catchment area). Eligible participants identified through the BCHD HIV surveillance system as part of standard BCHD linkage operations will be offered enrollment into the study during the linkage to care encounter.
- Pragmatic implementation trial with outcomes (such as retention in care) could be influenced by frequent study-related interactions; propose to minimize study contact to a baseline visit. During the baseline visit, the LTC officer will obtain basic demographic and contact information as routinely done for LTC operations.
- Participants randomized to the intervention arm will be given an option to get a study phone with a pre-paid data plan or, if they own a smartphone, to be compensated for their data plan so that they can download and use the mHealth application. Investigators have shown feasibility of this approach even with unstable populations such as people who inject drugs in Baltimore
- Because outcome of interest (virologic suppression, retention in care) could be influenced by monetary incentives, no monetary incentives during the study, except for completing client satisfaction surveys. The incentive structure will be discussed with the Steering Committee and adjusted based on their recommendations if needed.
- The mHealth application is designed to facilitate communication between the patient and the linkage officer. It also automates certain tasks/alerts/reminders, so that linkage officers can focus their efforts on patients at high risk of falling out of care (such as calling a patient with a high viral load), rather than on time-consuming tasks not specifically targeted to high risk patients (like general appointment reminders).
- Test for the equality of proportions of virologically suppressed/not suppressed or missing VL individuals at 12 months between the two study arms, in line with the randomized design of the study. Secondary to this, to adjust for any residual confounding, a logistic regression using individual outcomes (yes/no suppressed) with study arm as a main predictor and adjusting for patient demographic characteristics, socio-economic status at baseline, and other factors.
- A subgroup analysis will be conducted to assess the effects of the intervention among African-Americana and people of Hispanic/Latino descent.
- Power and sample size is well-justified, and accounts for potential withdrawals
- Quantitative and qualitative methods will be used to assess the mLTCR implementation processes over the follow-up period. Implementation will be evaluated in terms of core factors in the RE-AIM approach (hybrid method) for implementation science and informed by concepts from the IMB theoretical framework. Excellent methods.
- Interviews with linkage offers, in-depth interviews with participants randomized to intervention and control arm, client satisfaction surveys
- Limitations with pragmatic study design well-articulated (p 12) and the choice of utilizing a pragmatic study design is well-justified and rationalized.
- Relying on HIV surveillance data to assess outcomes. Primary outcome measure (documented VL < 200) may underestimate virologic suppression since some patients who do not have not had a viral load within the time frame may still be suppressed (but will be categorized as not suppressed). Underestimates of true virologic suppression will impact both study arms equally.
- Outcome definition of virologic suppression is consistent with definitions of virologic suppression used by the BCHD and MDH (basis for power calculations).
- Implement processes (training, weekly meetings, and performance monitoring) to optimize intervention fidelity.

Weaknesses:

- Because this is a pragmatic study, there is no routine study follow up visits and HIV surveillance data will be used for outcomes measures. Planning to enroll additional participants to account for an estimated 10% study withdrawal rate.

Criterion 4: Investigator(s) and environment

Reviewer 1:

Strengths:

- Dr. Page is an Associate Professor in the JHU School of Medicine. She has 10 years of experience working with HIV control problems in Baltimore's diverse areas.
- Other team members have expertise in implementation science (Dr. Chang), HIV and public health program implementation and evaluation (Dr. Page, Dr. Greenbaum, Glen Olthoff), mHealth interventions and evaluations (Dr. Chang and Jane McKenzie White), and qualitative research (Dr. Grieb).
- Partners at the BCHD also have the expertise they will need to be full partners in the scientific aspect of this effort.
- In all, this is an excellent research team.

Weaknesses:

- It is not clear to what extent the JHU researchers themselves reflect the diversity of the populations they will work with, which may present somewhat of a barrier to their work. Nevertheless, their cumulative experience in minority health plus the fact that BCHD partners are diverse should be sufficient to overcome this.

Reviewer 2:

Strengths:

Weaknesses:

Reviewer 3:

Strengths:

- This project is a collaborative study between the Baltimore City Health Department (BCHD) and Johns Hopkins University. Two PIs will serve as co-PIs for this proposal. Both PIs are medical faculty at Johns Hopkins and are uniquely qualified and have the experience and credentials needed to conduct the proposed study.
- The application offers a rationale for having two co-PIs and the leadership plan adequately describes PI roles and areas of responsibility: One PI has expertise in implementation science that will complement the other's close working relationship with BCHD, allowing for application of appropriate study design and evaluation frameworks for this proposal. HIV/AIDS outcomes.
- (Major) The application provides sufficient evidence that the PIs, collaborators, and other researchers involved in this proposed project have the clinical, community-based research, cultural, and statistical expertise to conduct the study. The PI is uniquely qualified to lead the team. She is Chief of HIV/STD/TB Clinical Services for the BCHD and the Johns Hopkins Center for AIDS Research (CFAR) Baltimore Core Leader. She has established multiple programs focused on decreasing the incidence of STIs in high-risk populations and on improving HIV and HCV outcomes through individual and structural interventions. Through participatory community processes and peer-driven health interventions, her tea, has have successfully engaged newly arrived Latino immigrants at risk of HIV infection, commercial sex workers, and African American men who have sex with men into care. The PI is also currently funded by the NIH (R01) to conduct a cluster- randomized trial in Baltimore City evaluating the impact of a mobile clinic that provides integrated health services in improving HIV outcomes among people who inject drugs.

- The BCHD support is appropriate for the proposed research. The institution (Johns Hopkins) provides adequate resources and support for the study, as demonstrated by letters of support.

Weaknesses:

- None noted.

<u>Reviewer 4:</u>

Strengths:

Weaknesses:

<u>Reviewer 5:</u>

Strengths:

- Dual PIs (Drs. Page and Chang) with complementary expertise; track-record of funded research; clear and well-delineated leadership plan
- Well-rounded team supporting the PIs with needed experience and evidence of strong capacity
- Unique partnership and collaborative research study between the Baltimore City Health Department (BCHD) and Johns Hopkins University.
- The study team has experience with cultural and linguistic adaptation of interventions and will elicit feedback from the Steering Committee
- Johns Hopkins University is ideally-suited to lead this research
- Conducting this study in Baltimore is a strength, given that African Americans have five times the rate of HIV infection compared to whites, and account for 78% of all new infections

Weaknesses:

- None noted.

Criterion 5: Patient-centeredness

<u>Reviewer 1:</u>

Strengths:

- This study was conceptualized from discussions with patients, providers, linkage officers and BCHD officials regarding barriers and facilitators to engagement in HIV care, and ways to leverage technology to facilitate provider-patient communication. Extensive discussions with patients and other stakeholders during the planning phase of this proposal reportedly "already led to important modifications."

Weaknesses:

- This patient population represents an extremely "hard to reach" group, which may limit their engagement in their treatment options and the direction of the overall study. This is because they are not clearly linked with the

system, so this makes this problem a very minor one.

Reviewer 2:

Strengths:

- The selected intervention for this study provides opportunities for HIV-infected patients to develop a trusting relationship through ongoing automated and direct communications with an assigned linkage officer who in-turn can respond specifically to their needs. These linkage officers will meet weekly with the research study team to discuss challenges observed among assigned patient participants in order to develop responses to these challenges. This is a MODERATE STRENGTH.
- The study research team will conduct in-depth interviews with participants to assess program fit and appropriateness of both interventions (one-time linkage services and the automated mHealth enhanced Linkage to Care and Retention 12-month smartphone application services) for satisfaction and recommendations. This is a MAJOR STRENGTH.
- The interventions selected for this application are available to patients now and will help them to receive communications that focus on issues related to HIV care as well as their patient-directed requests. These communications include medication reminders, pharmacy refills, scheduling appointments and facilitating transportation. This is a MAJOR STRENGTH.
- The mHealth enhanced Linkage to Care and Retention (mLTCR) intervention will link patients to resources that address their current social determinants of health including housing, substance use issues and intimate partner violence that are extremely important to patients and impact on their retention in treatment. This is a MAJOR STRENGTH.

Weaknesses:

- None noted.

Reviewer 3:

Strengths:

- The application describes how the intervention promotes active engagement with the patient from the planning through implementation and end-use (via technology).
- The proposed intervention to be tested has the potential to promote treatment adherence, better health outcomes, and a better quality of life for persons with HIV by sustained linkage to care.
- The control arm is being compared to the intervention is ‘treatment as usual’ and is currently available to patients now and is the best option for comparison. Patients randomized to the control arm will be linked to care by warm handoff per BCHD protocol (one-time interaction). Participants randomized to the intervention arm will be linked to care by warm handoff per BCHD protocol, but will continue to be in touch with the linkage officer for 12 months using the app, and by phone or in person, depending on the participant's needs (as identified through the app algorithms and patient-generated messages).

Weaknesses:

- None noted.

Reviewer 4:

Strengths:

- In the planning stages of this study, discussions were initiated with patients, providers, linkage officers and BCHD officials about the barriers to engagement in HIV Care and ways to facilitate communication between provider and patient to improve care retention. During these discussions, it was communicated that the most important outcomes were viral load suppression and the retention of care, which supports a demand for this kind of a study from end users. Major strength.
- The application has provided information that indicates that closing the evidence gap for disparities in HIV outcomes in minority communities is important for patients and other stakeholders. Moderate strength.
- The control arm leverages an existing public health infrastructure which is available to patients now. The intervention arm adds a longitudinal retention in care support through an mHealth application that is grounded in the Information-Motivation-Behavioral Skills (IMB) Model of Adherence, which is an evidence-based approach to understanding adherence to care. The long-term goal is to develop an intervention that improves HIV outcomes and can be integrated effectively into health department workflows. It is unclear at this point if this comparator would be chosen by patients but it seems that it would be a good choice for providers if the patients will use it. Moderate strength.

Weaknesses:

- None noted.

Reviewer 5:

Strengths:

- Research questions were developed with HIV providers, public health officials, linkage specialists, and people living with HIV: 1) Can longitudinal retention services provided by public health officers and facilitated by mHealth improve HIV outcomes and retention in care? 2) What is the feasibility and acceptability (from the patient, linkage officer, and health official perspective) of an mHealth-enhanced LTCR approach that leverages public health surveillance data and facilitates communication between linkage officers and patients?
- Outcome measures were simplified to include only two items that providers, public health officers and patients indicated to the investigators are most important to them: viral load suppression and retention in care.
- Study was conceptualized from discussions with patients, providers, linkage officers and BCHD officials regarding barriers and facilitators to engagement in HIV care, and ways to leverage technology to facilitate provider-patient communication.
- Extensive discussions with stakeholders during the planning phase of the proposal led to a pragmatic “real life” study so that the results can be generalizable to public health practice.
- Project will develop a phone App to provide additional support by allowing a secure way for patients and Linkage to Care Officers to communicate on an ongoing basis. The App was designed with patient input to make sure it was useful. Patients told researchers that it would be important for the App to help them make or reschedule appointments, get medication refills and send confidential reminders about upcoming appointments.

Weaknesses:

- Cost is anticipated to be a primary barrier to implement this research in other settings.
- Barriers to uptake of and usage of app could be articulated.

Criterion 6: Patient and stakeholder engagement

Reviewer 1:

Strengths:

- This is a collaborative study between the Baltimore City Health Department (BCHD) and Johns Hopkins University. It is clear throughout the proposal that BCHD is a true partner in this effort.
- Patients and other stakeholders will be represented on the steering committee.

Weaknesses:

- It is not clear whether being Spanish-dominant would limit participation as a stakeholder. Involvement of only bilingual or monolingual English speakers will limit the ability to get a true picture of this ethnic/language subgroup.

Reviewer 2:

Strengths:

- The research team has actively engaged HIV providers, public health officials, behavioral researchers, linkage specialists and people living with HIV to develop the concept for the mHealth LTCR intervention used in this study. These engaged stakeholders have been and will continue to be instrumental in examining the intervention's feasibility, acceptability, coverage, sustainability and perceived patient satisfaction in order to ensure that the study can be carried out successfully. This is a MAJOR STRENGTH.
- The Steering Committee for this study will engage African-American and Latino HIV-infected patients and outreach workers to help guide the study design, implementation, evaluation and dissemination of findings into the community. This Committee will additionally help adapt the mHealth intervention to ensure that its messages are tailored to the health literacy and language proficiency of the target population. This is a MAJOR STRENGTH.
- The Engagement Plan includes a mixed-methods (quantitative and qualitative data collection) study evaluation that will collect feedback from and engage the intervention's African-American and Latino HIV infected consumers at their Baltimore City Health Department utilizing their linkage to care (LTC) services. This is a MODERATE STRENGTH.

Weaknesses:

- The Steering Committee is scheduled to meet quarterly to review study progress. problem solve challenges and examine preliminary results which may prove too infrequent for real-world active engagement and decision-making authority by its patient members. This is a MINOR WEAKNESS.

Reviewer 3:

Strengths:

- The applicants involved HIV providers, public health officials, linkage specialists, and people living with HIV to develop the research questions.

- The application provides a well-justified description of how the research team incorporates stakeholder involvement and application describes how the study was conceptualized via discussions with healthcare consumers, providers, linkage officers and BCHD officials about barriers and facilitators to engagement in HIV care, and ways to leverage technology to facilitate provider-patient communication.
- Discussions with stakeholders during the planning phase of the proposal led to critical modifications. For example, the county health department partners have expressed a strong preference for a pragmatic "real life" study so that the results can be generalizable to public health practice, and outcomes measures were simplified to include only two items that providers, public health officers and patients indicated are most important to them: viral load suppression and retention in care.
- The composition of the Study Steering Committee includes African American and Latino individuals affected by HIV, HIV clinicians and linkage officers, public health officials, and members of the non-profit sector.
- The frequency and level of patient and stakeholder involvement are sufficient to support the study goals. For example, during the preparation phase of the trial, the Steering Committee will meet at least monthly to provide feedback on the new intervention app to ensure that the messaging is appropriate, clear, and the applications for patients and linkage officers are easy to navigate and use.

Weaknesses:

- None noted.

Reviewer 4:

Strengths:

- The study was developed from discussions with patients, providers, linkage officers and BCHD officials. These stakeholders have been involved during the planning phase, and their input led to modifications in the study design. Major strength.
- The roles and decision making responsibilities of the co-PIs and the 15 members of the Steering Committee are thoroughly spelled out in this application. The Steering Committee will meet at least monthly to provide feedback on the application, review eligibility criteria, discuss survey questions and outcomes measures, and provide other perspective. The frequency and level of patient and stakeholder involvement is sufficient to support the study goals. Moderate strength.
- This study is a collaboration between a city health department (BCHD) and Johns Hopkins University, and the resources are appropriate for the study throughout the project. Moderate strength.

Weaknesses:

- The Steering Committee is composed of HIV-infected African American and Latino patients, Linkage Officers, disease intervention specialists, public health officials, a Ryan White representative, and non-profit sector representatives. Representation from payors would be beneficial to provide insight on the financial aspects of this interventions to help ensure sustainability should this intervention be shown to be effective. Moderate weakness.
- Patients who are randomized to the intervention arm will either get a study phone with pre-paid data or compensated for data costs if they own their own smartphone. There is no mention of what happens if the patient uses the data for reasons other than the intended use for this study, and runs out of data for the intended application. Minor weakness.
- The absence of monetary incentives to patients in this study (except for the qualitative assessments & client satisfaction surveys) may inhibit participation / study retention. Minor weakness.

Reviewer 5:

Strengths:

- Johns Hopkins and Baltimore Health Department have appropriate organization structure and resources to engage patients and stakeholders during the project.
- Steering Committee (n=15 individuals) will meet at least monthly to provide feedback on the mLTCR application to ensure that the messaging is appropriate, clear, and the applications for patients and linkage officers are easy to navigate and use. The Steering Committee will also develop an implementation toolkit for mLTCR services that can be adapted to other settings. The Steering Committee with have a dissemination subcommittee.
- The research team will employ an iterative process of dissemination throughout the project period, disseminating findings to all partners and stakeholders, including study participants, through multiple venues and in ways that are understandable, respectful, and useful.
- Other stakeholder groups will be engaged in evaluation efforts including linkage officers, clinicians, and public health officials at the BCHD.

Weaknesses:

- Not particularly innovative methods but solid for the project.

Does the application have acceptable risks and/or adequate protections for human subjects?

Reviewer 1:

Yes

Reviewer 2:

Yes
N/A

Reviewer 3:

Yes

Reviewer 4:

Reviewer 5:

Yes

Overall Comments:

Reviewer 1:

The study is being proposed by Professor Kathleen Page and her colleagues at Johns Hopkins School of Medicine and in the Baltimore County Department of Health. Dr. Page and her team propose a four year "pragmatic individual

randomized controlled trial" comparing standard Baltimore County Health Department (BCHD) linkage services to the experimental arm of an mhealth and LTCR services intervention.

The investigators make an excellent argument for this being a highly needed study given their emphasis on African-American and Latino HIV infected individuals who do not have regular linkages to the healthcare system. The primary outcome of the study is viral load suppression with the secondary outcome being retention in care. The investigators have contracted with an expert corporation in mHealth and have experience with many aspects of this and related intervention.

There are several concerns about the overall study, however. The first and foremost of these limitations is the potential expansiveness of mHealth approaches especially with this population, possibly limiting its adoption. The diversity in the target population represents a potential scientific concern given that it would involve eligible individuals who are 18 and older, Latino or African-American, and men or women. Although the N of 500 seems adequate for overall effect analysis, it is not clear whether there would be enough power to look at any specific subgroup analysis. In all, strengths greatly outnumber weaknesses, and whatever weaknesses there is stem largely from the incredible challenge that these investigators are taking on.

Reviewer 2:

This application proposes a "real-world" pragmatic randomized controlled study addressing health disparities of public health treatment services for African-American and Latino HIV infected patients in an urban setting. The assembled research team has years of experience engaging community stakeholders and developing health programs to specifically address HIV among minority low-income populations served in Baltimore City. The primary intervention utilizes two (2) smartphone mobile applications designed to: (1) help facilitate communications between HIV infected patients and outreach workers, (2) address social determinants of health faced by low-income HIV infected patients and (3) significantly improve HIV treatment outcome measures on virologic suppression and retention in care.

One of the unique elements of this study is that the research team is aware of the challenges and barriers faced by this patient population and are willing to address them by actively engaging end-users and key stakeholders throughout the study process. The proposed mHealth-enhanced Linkage to Care and Retention (LTCR) intervention will help facilitate patient-directed and treatment focused communications with outreach workers who act as linkage officers to patient requested resources, answers and support. Noteworthy is that "real-life" social determinants of health such as housing/homelessness. Intimate partner violence and substance abuse often faced by this study's population are identified and addressed as barriers and challenges to patient treatment retention.

The Study Plan and the Engagement Plan in this application are well thought out and clearly designed to be patient-centered and practical in an urban public health setting. As a result, study findings appear to be generalizable with the potential to inform clinical practice at health departments serving HIV infected low-income patients across the country. The only concern is that this study's findings are site-specific to one Baltimore City Public Health setting which may or may not prove significant enough to impact health policy and treatment services in other public health settings across the country.

Reviewer 3:

This study strives to ascertain if longitudinal retention services provided by public health officers and facilitated by mHealth can improve HIV outcomes (virologic suppression) and retention in care (at 12 months) as well as assess the intervention's feasibility, acceptability, fidelity, reach, sustainability, and adherence (from the patient, linkage officer, and health official perspective) of an mHealth-enhanced linkage to care (LTCR) approach that leverages public health surveillance data and facilitates communication between linkage officers and patients. The mHealth LTCR (mLTCR) intervention consists of two smartphone applications, one for patients and one for linkage officers (community members), to help facilitate communication. Communication will focus on issues related to HIV care (e.g., appointment scheduling, medication reminders, refills, transportation), and patient-directed requests.

Using HIV surveillance data (e.g., unsuppressed HIV viral load), linkage officers will be automatically alerted if a patient had a high viral load and prompted to contact the patient. In addition to appointment and medication reminders, patients will receive positive reinforcement behavioral text messages. The mLTCR protocol will also include options to link to resources that address social determinants of health, such as housing, substance use disorder, and intimate partner violence. This study aims to provide evidence to discover if 1) longitudinal retention services provided by public health officers and facilitated by mHealth can improve HIV outcomes and retention in care, and 2) the feasibility and acceptability (from the patient, linkage officer, and health official perspective) of an mHealth-enhanced LTCR approach that leverages public health surveillance data and facilitates communication between linkage officers and patients.

Providing linkage and longitudinal retention (LTCR) services from the health department that complements existing clinic-based retention efforts has not been systematically evaluated. Therefore, this study has the potential to benefit stakeholders on all levels. The applicants assert that increased contact with a clinic can improve retention in care for persons with HIV but may not be enough for patients with other unmet needs. The applicants state that providing longitudinal support may enhance timely linkage to care and facilitate future re-engagement attempts, if needed, particularly with a highly tailored technology-driven intervention such as the one proposed here.

The application describes a pragmatic study that will make inform decision-makers as the protocol will be replicable in other public health settings similar to the Baltimore County Health Department (BCHD). It utilizes the IMB Skills Model of Adherence, which has been proven effective for persons living with HIV/AIDS, highly appropriate for a study that aims to inform, motivate and engage patients for positive change (e.g., adherence). Possible modifying factors affecting adherence (substance use, housing, insurance coverage) are included (yet not integrated) in the model. However, cultural, racial, and linguistic factors are insufficiently discussed as possible modifiers. It is likely if the intervention is effective it could be reproduced by other county health departments to promote better patient outcomes. The application makes a cogent case for the importance of linking HIV-infected patients out of care or newly diagnosed to services who are at risk for low or no engagement with the healthcare system.

The team is experienced and well-poised to conduct the study. The frequency and level of patient and stakeholder involvement is sufficient to support the study goals. A Steering Committee with existing and new partners, including African American and Latino patients, healthcare providers, outreach workers, and representatives of the non-profit sector are sufficiently involved in modifying the mHealth application as needed and guide the study design, implementation, evaluation, and dissemination of findings to the community.

The application describes the conceptual framework, IMB-skills model to inform the design, key variables, and relationship between interventions and outcomes being tested; it is appropriate for this study. Sample sizes and power estimates are appropriate for this proposed RCT, and the project timeline is feasible. However, the research methods have some notable weaknesses.

The application needs a more detailed explanation regarding qualitative data analysis and the process by which a mixed methods approach will be undertaken. It also does not adequately describes the rationale for a grounded theory approach over other forms of qualitative analytic approaches. The application does not provide an interview guide for the qualitative interviews and how they intend to provide interviews in Spanish if needed. Regarding linkage officer abilities, it is unclear how variations in linkage officers' abilities, experience, and training in this practice could affect study outcomes; proposal does not thoroughly discuss how those linkage officers will be trained and how/whether their interactions with patients will be standardized or monitored. Moreover, there is no mention of accounting for other confounders, such as a physician or other clinic staff influences in patient engagement.

The application utilizes the IMB Skills Model of Adherence, which has been proven effective for persons living with HIV/AIDS, highly appropriate for a study that aims to inform, motivate and engage patients for positive change (e.g., adherence). Possible modifying factors affecting adherence (substance use, housing, insurance coverage) are included (yet not integrated) in the model. However, cultural, racial, and linguistic factors are insufficiently discussed as possible modifiers.

Overall, this is a strong application. The outcomes of this study (virologic suppression, linkage and retention in care), have the potential to improve clinical outcomes (improved survival and lower morbidity) and reduce HIV transmission in communities most affected by HIV. The information will also be relevant to Ryan White officials responsible for allocating funding to support clinical and ancillary services to improve HIV outcomes. Evaluating this intervention via a local health department as proposed by the application will increase the generalizability of the results and has the potential to inform practice at other health departments. However, a major challenge (and concern) of this intervention that requires thoughtful consideration is the burden of its cost that may not be feasible at the nonprofit community health agency level. It would behoove the applicant to offer some more economical alternatives to the Emocha platform and/or discounts for cell phone data usage.

Reviewer 4:

This application has given sufficient background information to create a compelling rationale for the need for this type of study.

The major strength of this study is the collaboration between Johns Hopkins University and the Baltimore City Health Department. The research plan would be to develop an intervention to help patients stay in care to improve their HIV outcomes and to test this intervention within an existing public health infrastructure. This could increase the likelihood of this intervention being adopted in other public health systems if found to be effective.

- There are several weaknesses to this application, but they are mostly fixable. Having payors represented as key stakeholders (or on the Steering Committee).
- Provide a more detailed Engagement Plan for patients and stakeholders.
- Start the dissemination plan earlier in the project.

Reviewer 5:

Leveraging mHealth and peers to Engage African-Americans and Latinos in HIV care (LEAN):This is well-written and conceptually-sound grant application representing a multi-sector partnership between Johns Hopkins University and the Baltimore City Health Department. The overall goal is to evaluate whether a Baltimore City Health Department (BCHD) mHealth-enhanced Linkage to Care and Retention (mLTCR) intervention can improve HIV outcomes among HIV-infected African American and Latinos compared to standard BCHD linkage to care protocols. The project proposes a 4-year pragmatic individual randomized controlled trial comparing standard BCHD linkage services (control) to mHealth-enhanced LTCR services (intervention). It proposes to enroll 500 (250 per arm). The primary outcome is viral load suppression (< 200 copies/ml) 12 months after study enrollment. The secondary outcome is retention in care. Secondary outcomes include 1. Time to virologic suppression, 2. Time to linkage to care, 3. Retention in HIV care, and 4. Patient satisfaction. HIV-infected adults (≥ 18 years of age) identified through the BCHD HIV surveillance to be newly diagnosed or out-of-care will be eligible for participation in the study and offered enrollment during the linkage to care encounter. The intervention is grounded on the Information-Motivation-Behavioral Skills (IMB) Model of Adherence, an evidence-based and theory-driven approach to understanding adherence to medical care. The intervention is well-described in the tables and mapped onto the theoretical framework. In the control arm, BCDH linkage officers contact out-of-care patients by phone or in person and provide a warm handoff and same-day appointment to the clinic of the patient's choice. The intervention arm includes longitudinal retention in care support with the aid of an mHealth application that facilitates ongoing communication between the patient and the linkage officer. A mixed-methods evaluation will be conducted to examine the implementation of mLTCR services (qualitative interviews and surveys) using the RE-AIM approach for implementation science. Key strengths: Interdisciplinary team; JHU and department of health multi-sector collaboration; steering committee with key community partners; leverages existing structures (current BCDH linkage program and officers) and enhances using technology mLTCR intervention (potential for scalability); a subgroup analysis will be conducted to assess the effects of the intervention among African-Americans and people of Hispanic/Latino descent. Weaknesses: The investigators indicated the population focus: Adults 21-64; Racial or ethnic minorities; Residents of

urban areas; Low-income groups Project primary focus is HIV-infected African Americans and Latinxs, which will include some people with limited English proficiency and members of the LGBTQ community. However, there was no substantive/ meaningful mention of the LGBTQ community in the application.
